# Population Dynamics of Early Human Migration in Britain

**DOI:** 10.1371/journal.pone.0154641

**Published:** 2016-05-05

**Authors:** Mayank N. Vahia, Uma Ladiwala, Pavan Mahathe, Deepak Mathur

**Affiliations:** 1 Tata Institute of Fundamental Research, Homi Bhabha Road, Mumbai 400005, India; 2 UM-DAE Centre for Excellence in Basic Sciences, University of Mumbai, Kalina, Mumbai 400098, India; Universitat Pompeu Fabra, SPAIN

## Abstract

**Background:**

Early human migration is largely determined by geography and human needs. These are both deterministic parameters when small populations move into unoccupied areas where conflicts and large group dynamics are not important. The early period of human migration into the British Isles provides such a laboratory which, because of its relative geographical isolation, may allow some insights into the complex dynamics of early human migration and interaction.

**Method and Results:**

We developed a simulation code based on human affinity to habitable land, as defined by availability of water sources, altitude, and flatness of land, in choosing the path of migration. Movement of people on the British island over the prehistoric period from their initial entry points was simulated on the basis of data from the megalithic period. Topographical and hydro-shed data from satellite databases was used to define habitability, based on distance from water bodies, flatness of the terrain, and altitude above sea level. We simulated population movement based on assumptions of affinity for more habitable places, with the rate of movement tempered by existing populations. We compared results of our computer simulations with genetic data and show that our simulation can predict fairly accurately the points of contacts between different migratory paths. Such comparison also provides more detailed information about the path of peoples’ movement over ~2000 years before the present era.

**Conclusions:**

We demonstrate an accurate method to simulate prehistoric movements of people based upon current topographical satellite data. Our findings are validated by recently-available genetic data. Our method may prove useful in determining early human population dynamics even when no genetic information is available.

## Introduction

Extracting comprehensive data relating to prehistoric human settlements is notoriously difficult as the available evidence is, more often than not, sparse, scattered, perishable, and prone to misinterpretation. It is, therefore, of obvious utility to develop new and independent methods of study that may not only provide insights into prehistoric population dynamics but also help predict potential locations of prehistoric settlement sites.

Prehistoric human migrations may be effectively correlated to parameters that quantify availability of essential resources [1,2]; relatively few migrations are based upon risky explorations initiated for frivolous reasons like adventure. Although early human migrations may be presumed to be largely driven by available resources, later migrations into areas already occupied are far more complex as they involve aggressions and warfare brought about by struggles between emerging groups and classes. A generalized approach to human migration is, thus, complex and involves including both deterministic and stochastic processes [3,4].

Simulations of human migration necessitate taking into account not only the availability of resources essential to survival, but a host of other complex factors: technologies for travel and transport, the nature of human social organisation, and the relative sizes of migrating populations and their interrelations [2]. Models attempting to account for all, or most, of these parameters are complex and are likely to include subjective criteria. Rice and Papadopoulos [4] have incorporated both deterministic and stochastic aspects of human migration to derive an exact equation for directional evolution in an open population. They have shown that increasing variance in migration rates reduces the impact of migration relative to selection based on phenotypes. Hence, large-group migrations are fundamentally different from small-group migrations. Models that treat migration as a single parameter are expected to overestimate the impact of immigration on the resident population.

Rice and Papadopoulos [4] have, further, shown that selection and migration interact in complex ways and that the role of migration in evolution is determined by the entire distribution of immigration and emigration rates, not just by their mean values. The interactions of stochastic migration with stochastic selection produce evolutionary processes that may become obvious in deterministic evolutionary theory.

Early human migrations into hitherto unoccupied territory may be modeled more simply by identifying regions with superior resource availability. These populations tend to seek availability of water [5], food, shelter from predatory animals and avoidance of conflict with other human groups, as well as the physical comfort of relatively flat land. A moderate climate, and food provided by hunting game and foraging wild fruits and plants influences migration, at least until the establishment of an agrarian culture. Settlements would be spread over a fairly large area, with a moderate population density. Large habitations, such as cities, were unusual. Sometimes, availability of rock suitable for making tools may have become a determinant in the formation of such settlements. Petraglia and Allchin [6] have discussed these issues in detail in the context of South Asia.

A way to model very early human migration into more or less virgin territory would treat geography (mostly flat land and accessible water resources) and the availability of game and foraging opportunities as the primary deterministic factors. Verifying the validity and accuracy of such a model would, of course, be difficult. However, with recent availability of large-scale genetic data from relatively isolated populations may open possibilities of evaluating such models. We have developed a simplified model of population diffusion based on availability of natural resources and a suitable environment, with emerging data being amenable to comparison with genetic data. Our results offer useful glimpses into how initial populations may diffuse into new territories. We use the example of the United Kingdom since its islanded landmass has had less population mixing in the course of prehistory, as may be gleaned from recent, large-scale genetic data [7]. The results of our study pertaining to the United Kingdom have wider implications: they suggest that the combination of a deterministic simulation technique with genetic data may be of general utility in quantitatively determining population migrations pertaining, especially, to the prehistoric period. Our method is readily implementable as it uses satellite-based geographical information; it can provide insights into early human population dynamics even in the absence of genetic data.

## Method

### The simulation process

An important prerequisite of prehistoric population dynamics studies is availability of ancient geographical data, particularly of water bodies. Contrastingly, for our study which spans *ca*. 10,000 years before the present era, current geographical data appears to be sufficient as timescales for geological changes are considerably longer. In the present population dynamics study we utilize 1 km resolution satellite maps of the surface of the Earth (GLOBE 1 km Digital Elevation Model from NOAA—the National Oceanic and Atmospheric Administration—http://www.ngdc.noaa.gov/mgg/topo/globe.html) along with high accuracy hydro-shed data (http://hydrosheds.cr.usgs.gov/dataavail.php) for mapping water resources. Although higher resolution (30 m) satellite data were easily available, we found it far easier to use the 1 km-resolution data for our computations, without significant changes in overall accuracy.

We divided the entire stretch of the main island of Britain—comprising England, Scotland and Wales—into 1 km x 1 km square segments. These were the primary units for our computations. We defined the habitability of each segment by four parameters: altitude, surface-kind (a parameter of relative flatness), proximity to a water source, and population density. We used an agent-based, finite-element diffusion model of population migration across the island. Details of our algorithm are presented in the Appendix.

In agent-based models the fixed geography-dependent parameters, like altitude, surface-kind, and water-source proximity, are referred to as patches while population is denoted as an agent. Population density would be neither an agent nor a patch. Altitude, denoted as D_alt_, can be related to the integrated population density which decreases faster than exponentially as the altitude increases from sea level to ~1000 m; thereafter, it rises again, albeit at a slower rate, to peak at ~2300 m before falling off again [8] with a cut-off at 4100 m. Surface-kind is different from altitude in that it considers the gradient of the land. In the context of the British Isles, it is assumed that, even if a location is not at a high altitude but is sloping, it is less desirable. Humans tend to adapt to sloping land by cutting into or flattening small portions. Surface-kind, D_surf_, therefore, has a relatively weak dependence on the diffusion process. The water-source parameter, D_riv_, is self-explanatory and is based on the work of Kummu et al. [5]. The population density parameter has been initially given externally. A parameter called maximum population density is defined that gives the maximum population density a region can sustain; it is based on values of the first three parameters.

Population movements into or out of any region are determined by the rates of emigration and immigration, which, in turn, are governed by the values of our four parameters. Together, they define the desirability of a piece of land. If the population exceeds even by a fraction of the maximum sustainable population, emigration rates increase significantly whereas immigration ceases until the population reduces significantly below the maximum sustainable population. Values of the population density parameter are taken to range from 0 to 1 for any land mass and are -1 for a water source. A linear combination of the four parameters is used to determine the absolute or population-independent rating of a given location, which we denote as R_in_. Rating changes dynamically with the population of a place, and equals R_*in*_ whenever the population is 0.

**Fig 1:**
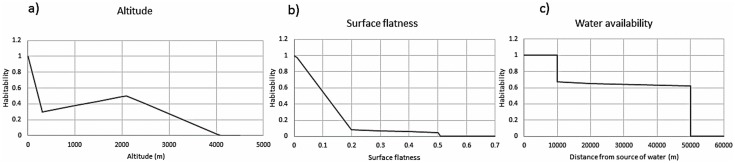
Habitability of a piece of land as a function of altitude, surface flatness, and the availability of water. The altitude sensitivity is the strongest. However, since many ancient cites are known to be located well above sea level, we have kept the habitability at relatively high values up to an altitude of 2 km. Land unevenness also has significant impact on choice of habitation; it exhibits a steeper function vis-à-vis habitability. The dependence on water is also based on experiences from early cultures [5]. We assume that up to 2 km distance, water can be directly accessed (carried) while, for distance beyond that, the permeability of land to create subterranean water sources assumes more importance. We, therefore, assume that access to water becomes difficult up to 10 km and falls with a power law between 2 and 10 km. Please see the text for more details.

The suitability of a location is quantitatively described in terms of normalized parameters listed above, and is called desirability. Thus, there is desirability that is based on altitude, surface-flatness, and water-proximity (Fig 1).

#### Desirability based on Surface Kind: D_surf_

The mean altitude of a place (A_mean_) was calculated by using the NASA database. Surfaces were designated flat, tolerable, or intolerable (too steep, for instance, or undulatory). Surface-kind depended on the deviation *d* of a location from the region’s mean altitude. If *d* was less than a certain L_flat_ (nominally set at 0.25), the surface was designated flat and the value of D_surf_ fell from 1 in a power-law. If *d* was greater than L_flat_ but less than L_tol_, the surface was designated tolerable and D_surf_ fell much more rapidly. Every other location was assumed to be uninhabitable and D_surf_ was then assigned a value 0.

#### Desirability based on location: D_loc_

The parameter D_loc_ is a linear combination of D_alt_ and D_surf_ with D_alt_, with a weightage of W_flat_.

#### Desirability based on water-source proximity

There are three possible approaches:

1. D_riv_ and distance to the water source follow an inverse square law. Any and every water source lying within a to-and-fro distance of 10 km is cosnidered acceptable.
2. D_riv_ and distance to the water source follow a linear square law over the same range as above.
3. D_riv_ follows the same relations as (1) but has double the range.

#### Rating of each site R_in_

The population independent rating of each site (R_in_) was calculated as a linear combination of D_riv_ and D_loc_. All flat land had R_in_ between 0.5 and 1. All tolerable land had R_in_ values of 0.25–1.

#### Optimum population of a location

The optimum population sustaniable in a location was the arithmetic mean of water-based optimum population and location-based optimum population. Both are a product of different parameters of desirability and P_max i_ for any location *i*.

#### Description of our simulation-engine

We further pre-computed values of R_in_ and optimum population (P_best_). The rating (R_i_) of location *i* was calculated as:

$$R_{i}\;=\;R_{in}+C\times P_{i}$$

where C is a constant and P_i_ is the currently existing population. C is nominally taken as 1.

In a given location, the rating-per-unit-population was calculated to be the parameter α (a linear combination of various desirability parameters). Migrating people tended to move along the direction with greater mean value of α. If we take the fraction of people moving towards a positive x direction as f_x_ and those moving towards positive y as f_y_, for a given direction and boundary, the difference of α’s is calculated to be δ. The polarization of migrating people towards opposite boundaries is defined as the function of two δ’s and is called β which is equivalent to the pressure “felt” by a boundary. The population moving towards a given boundary, the fraction of people moving in (F_i_) and out (F_o_), is defined as

$$F_{i}\;=\;f\times \beta$$

$$F_{o}\;=\;f\times (1-\beta )$$

Once these parameters are defined, the diffusion model is used with rating instead of pressure. We define an emigration rate E_i_ from a cell *i* as N_i_*R_i_. In a given boundary, N is the number of people moving towards the cell at the immigration rate (Ii). The number of people moving out is

$$I_{i}\;=\;\frac{\sum_{i}N\times R_{i}}{\sum_{i}R_{i}}$$

for *j* = 4 cells north, south, east and west

Hence, for every boundary we have emigration and immigration calculated as Em, Im or Em1, Im1 (for rows and columns). The net flux at any given boundary is Em-Im. The change in a cell is the net flux across all the four boundaries. The change matrix is computed in order to monitor emerging patterns. Population increase due to net birth is defined as 0.1*P_i_*R_i_.

## Results

### Habitability

**Fig 2:**
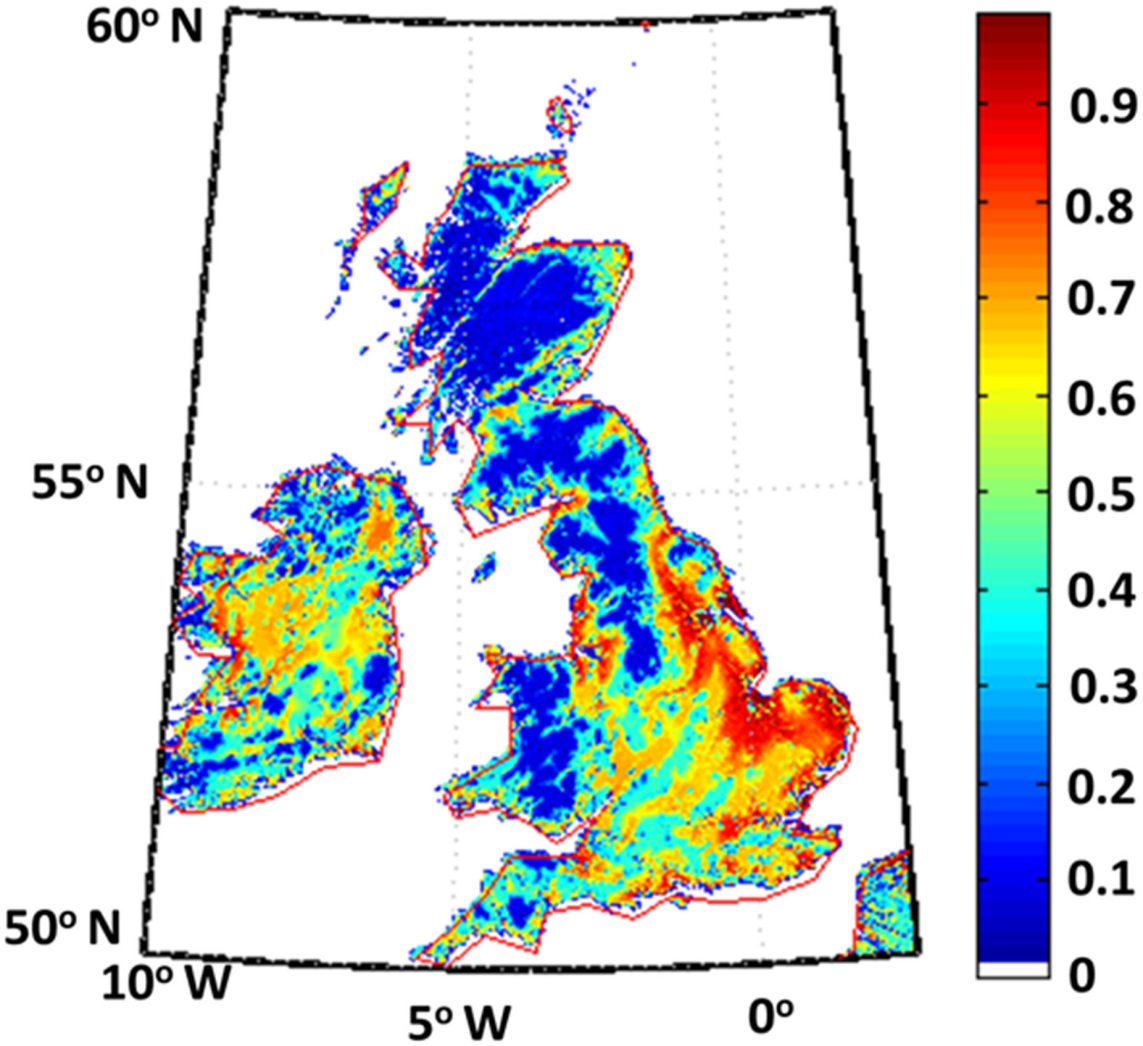
Habitability map of England, Wales, Scotland, and Ireland. The population density is highest in the red regions and is sparsest in the blue regions. The map corresponds to habitability before the entry of humans.

We have used the parameters described above to derive the habitability map of England, Wales, and Scotland (Fig 2). Our simulation treats humans as resource consumers possessing an affinity for good resources and with the pressure to migrate from any point to another point which depends on the

1. Population density in a given region, and
2. Habitability of the nearby zone.

### Entry points and diffusion of population

**Fig 3:**
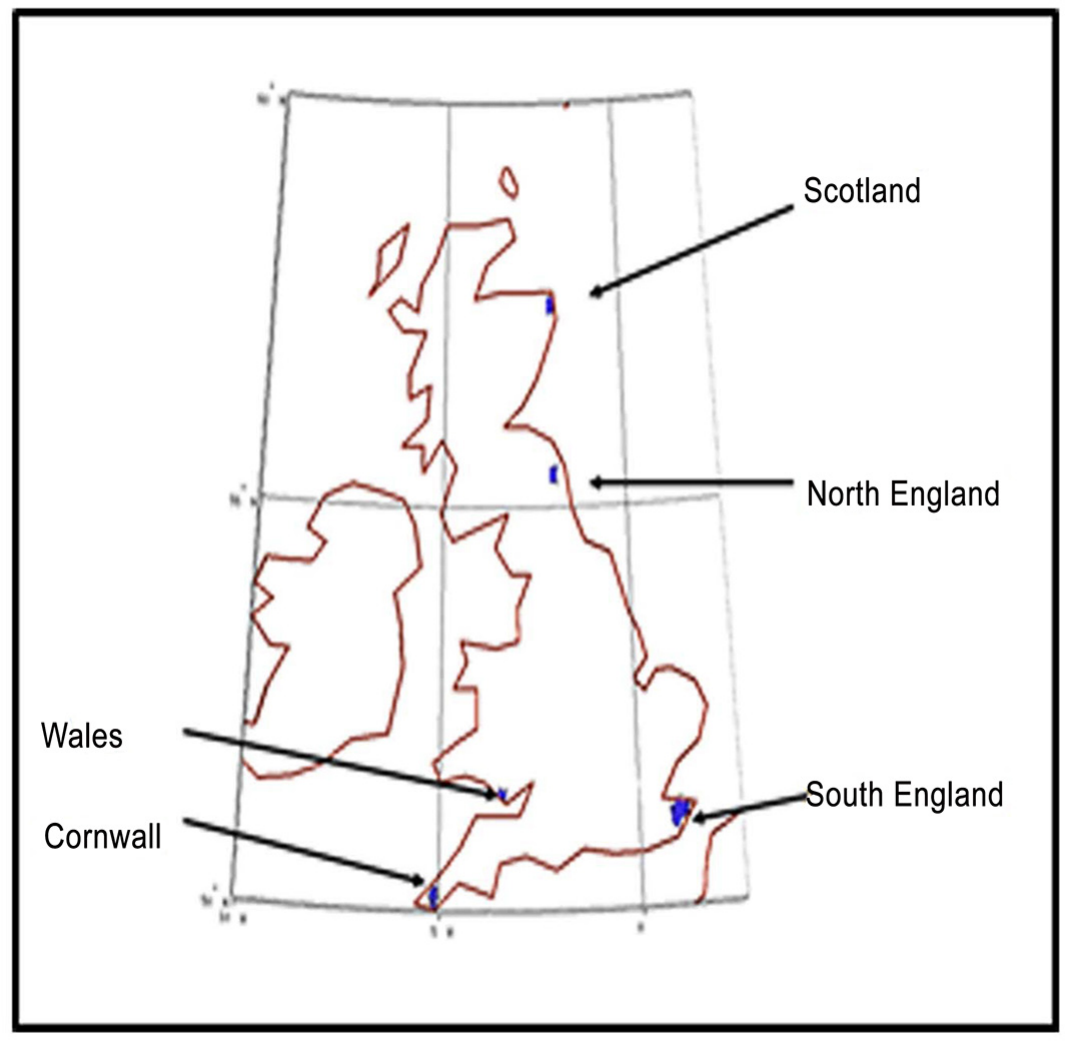
Locations of initial population in the simulation. The markers show the four locations from which the simulation begins.

In developing a desirability map we assume that people entered the main British island in five distinct groups at the following locations (Fig 3): Cornwall (50.1N, 5W), Wales (51.4 N, 3.43 W), Scotland (55.4 N, 2.0 W), North England (57.5 N, 2.0 W) and South England (51.1 N, 1.0 E) in the ratio of 10^3^:10^1^:10^1^:10^1^:10^5^. The ratios serve the purpose of simulating delayed entry of population at different points. Since the first entry is in South East England (see, for example, Figure 3 in [7]) we place the largest population in South England.

**Fig 4:**
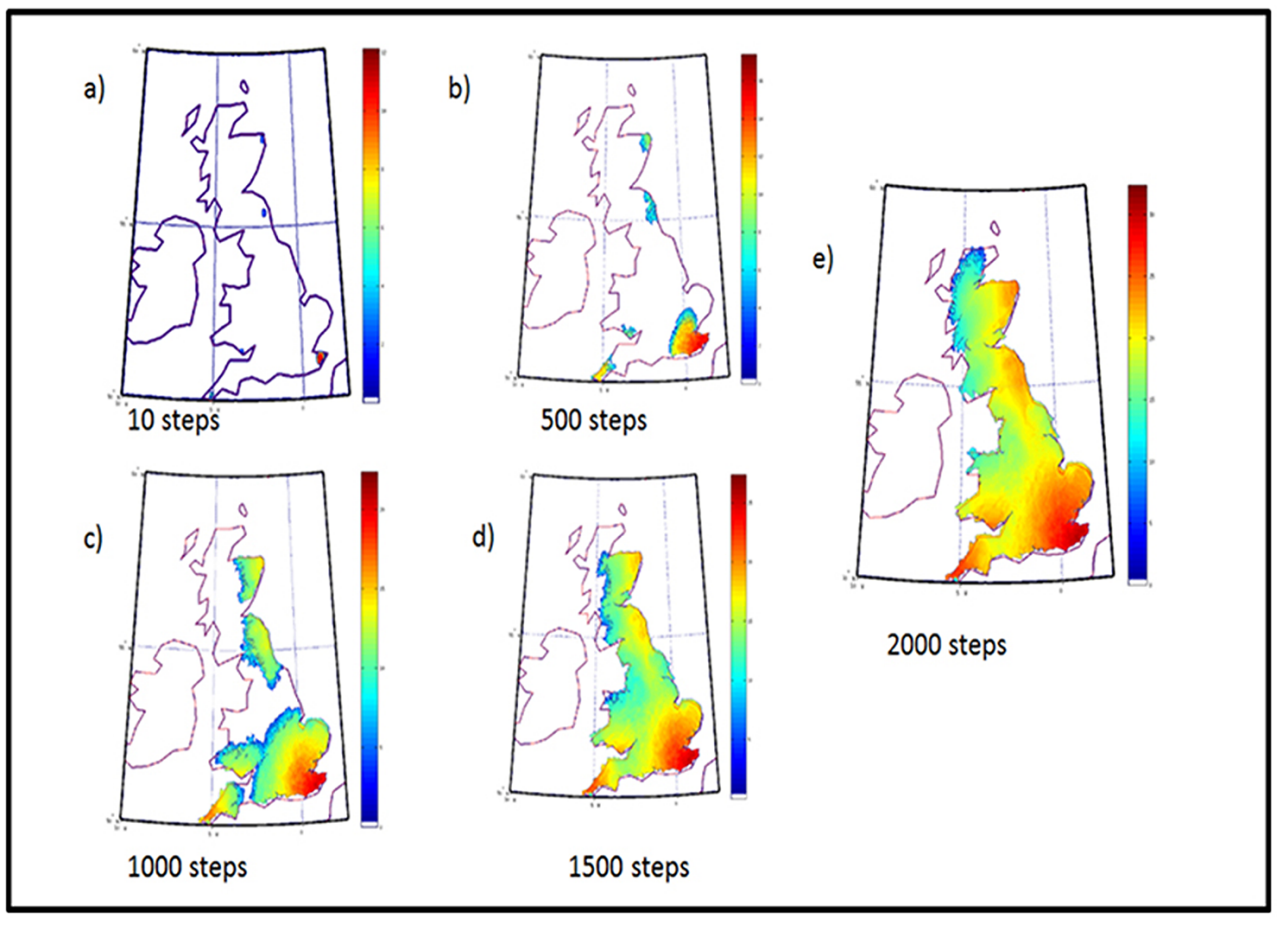
Evolution of the population pattern in steps of 500 iterations. Each image corresponding to roughly 500 years. The population density is highest in the red regions and is sparsest in the blue regions.

At each step our computer program checks the relative habitability of 4 squares (East, West, North, and South of the given location) surrounding a given square, and exchanges—imports or exports—people based on relative habitability (based on habitability and current population density). We then run the simulation for 500 steps at a time. In Fig 4 we present the results of our simulation after the first 2000 steps. Assuming that human drifts are at the rate of 1 km per year: each step amounts to about 1 year of diffusion.

Fig 4 shows the progressive evolution of population from the initial points of entry. Starting with isolated groups (Fig 4a), the population spreads at speeds that depend on local habitability (Fig 2). The rate of population increase that enforces movement is taken to be 0.1% of the population per cycle. During each simulation cycle, nine adjacent squares around each square are taken into consideration, and the population distributed on the basis of relative habitability. Fig 4c (1000 steps) shows the merger of the people of Wales with the population from South England and that of Scotland with North England. By the time 1500 years have elapsed, the populations are in contact with each other but it takes another 500 years to completely populate England. Since the simulation does not permit the population to cross a barrier of more than 25 km of sea, the population does not cross over into Ireland.

### Comparison with genetic data

**Fig 5:**
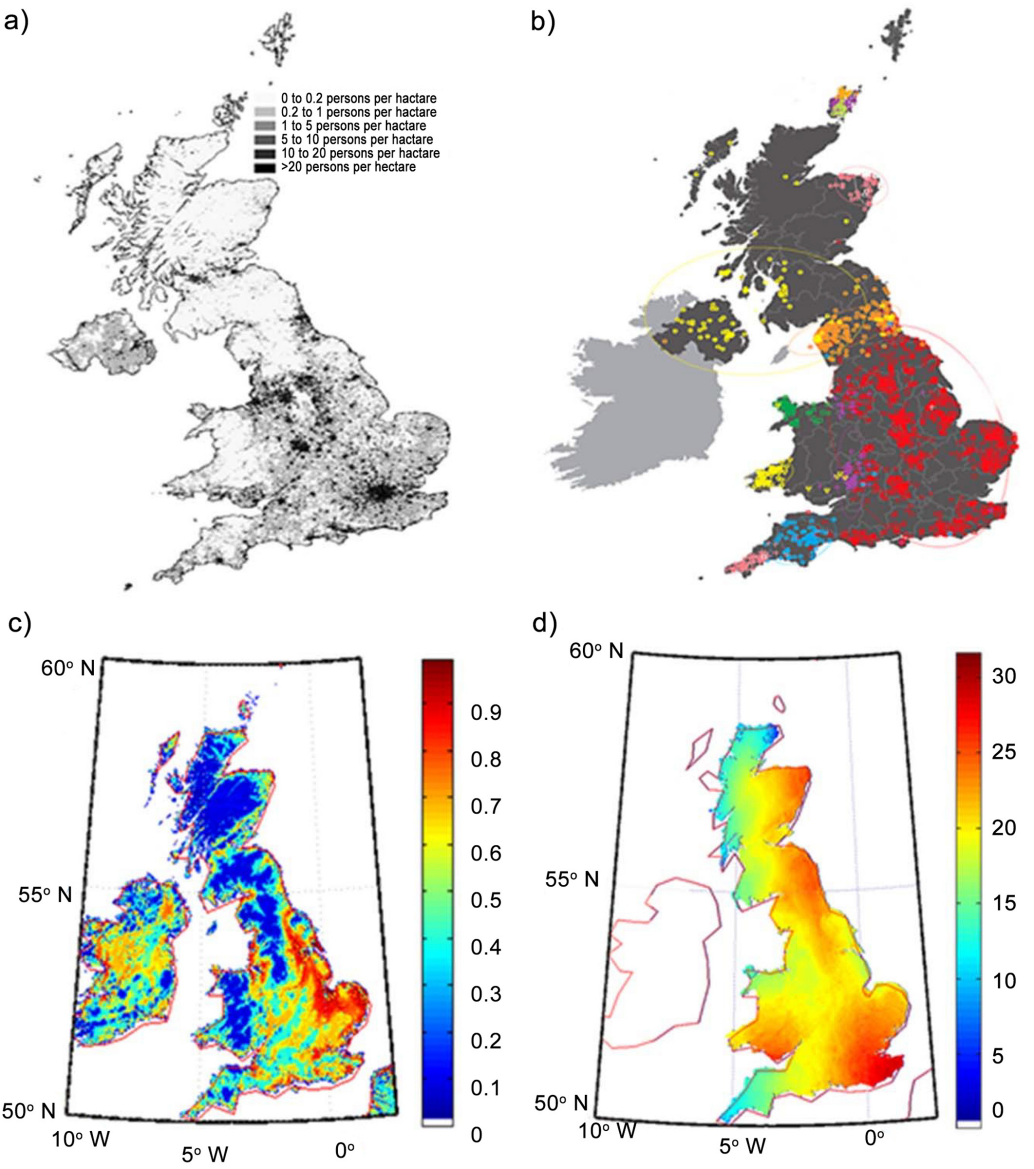
Maps of the British Isles. a) the current British population density b) the genetic map, c) the habitability of the landmass, and d) simulated distribution in population after 2000 steps.

Genetic profiling of human populations provides important information, including insights into demographics [9]. These have been documented both as broad-scale studies of defined locations [10, 11] or as fine-scale studies [12, 13]. In their recent study, Leslie *et al*. [7] have investigated the fine-scale genetic structure of a Caucasian population sample within the United Kingdom. Their results are schematically shown Fig 5b (figure 1 of [7]). They performed and analysed genome-wide autosomal single-nucleotide polymorphisms (SNPs) of 2039 sample subjects from rural UK, all of whom had all four grandparents born within an 80 km radius of each other. Since genetic differences could be related to migration and admixture of populations from outside the UK, they compared the information obtained with DNA samples of 6,209 Europeans. Their work confirmed, and in many cases shed further light on, known migration patterns. Using specific algorithms the samples could be grouped into hierarchical genetic clusters from coarser to finer levels, which were then mapped to geographical locations in the UK. Fig 5b reproduces the map for 17 such clusters [7].

A striking correlation was observed between genetic clusters and geography. Most clusters were localized and non-overlapping. At the coarsest level, the population in the Orkneys (islands to the north of Scotland) emerged as the most genetically distinct. At the next level, Wales formed a distinct genetic cluster, followed by a further division between north and south Wales. Northern England, Scotland, and Northern Ireland collectively appear separate from southern England, and Cornwall formed a separate cluster. Scotland and Northern Ireland were then differentiated from northern England. The largest single cluster comprising ~50% of the samples (red squares) covered central and southern England. The genetic differentiation appeared to follow geographical boundaries at times (Devon and Cornwall; Wales and England) and at times not (Northern Ireland and Scotland). This genetic profile was further correlated with major events in the peopling of the British Isles at different times (see figure 3 in [7]), based on archaeological, historical and linguistic evidence.

The analyses suggested that there was substantial migration across the English Channel after the original post-ice-age settlers, but before Roman times. The Welsh appear to have more similarities to the earliest settlers of Britain after the last ice age than do the other people in the UK. The single large cluster in central and southern England had significant DNA contribution from Anglo-Saxon migrations originating from European regions corresponding to present-day Germany, after the departure of the Romans.

Surprisingly, there was not a single, genetically distinct “Celtic” contribution in the non-Saxon part of the UK. In fact the Celtic parts of the UK (Scotland, Northern Ireland, Wales and Cornwall) were among the most genetically different from each other, as indicated in the hierarchical clustering tree in Fig 1 of Leslie *et al*. [7] (reproduced in Fig 4b). For example, the Cornish are genetically more similar to other English groups than to the Welsh or the Scots. No obvious genetic signature of Viking occupation was evident in spite of their control of large parts of England from the 9th century. A minor Norse contribution (~25%) was observed in the Orkney population. The study concluded that contribution of historical migration events on the genetic composition of the British Isles was less than would have been otherwise expected. One of the limitations of their study, as stated by the authors, was that although the genetic data enabled the inference of the relative order of migration events depending upon the extent of contribution to the clusters, it could not determine absolute times, nor distinguish between migrations of small numbers of people over longer periods or larger numbers over shorter periods.

## Discussion

We have made use of a diffusive model to define the habitability of different regions in Great Britain and have simulated the diffusion pattern of the population based on four entry points, 10,000 years BCE. While our simulation is sensitive to relative population density and entry points, the broad points of interaction are well defined and are based on the desirability map (Fig 1). The simulation also agrees well with the very recent genetic map of Britain, including figures 1 and 2 of [7].

**Fig 6:**
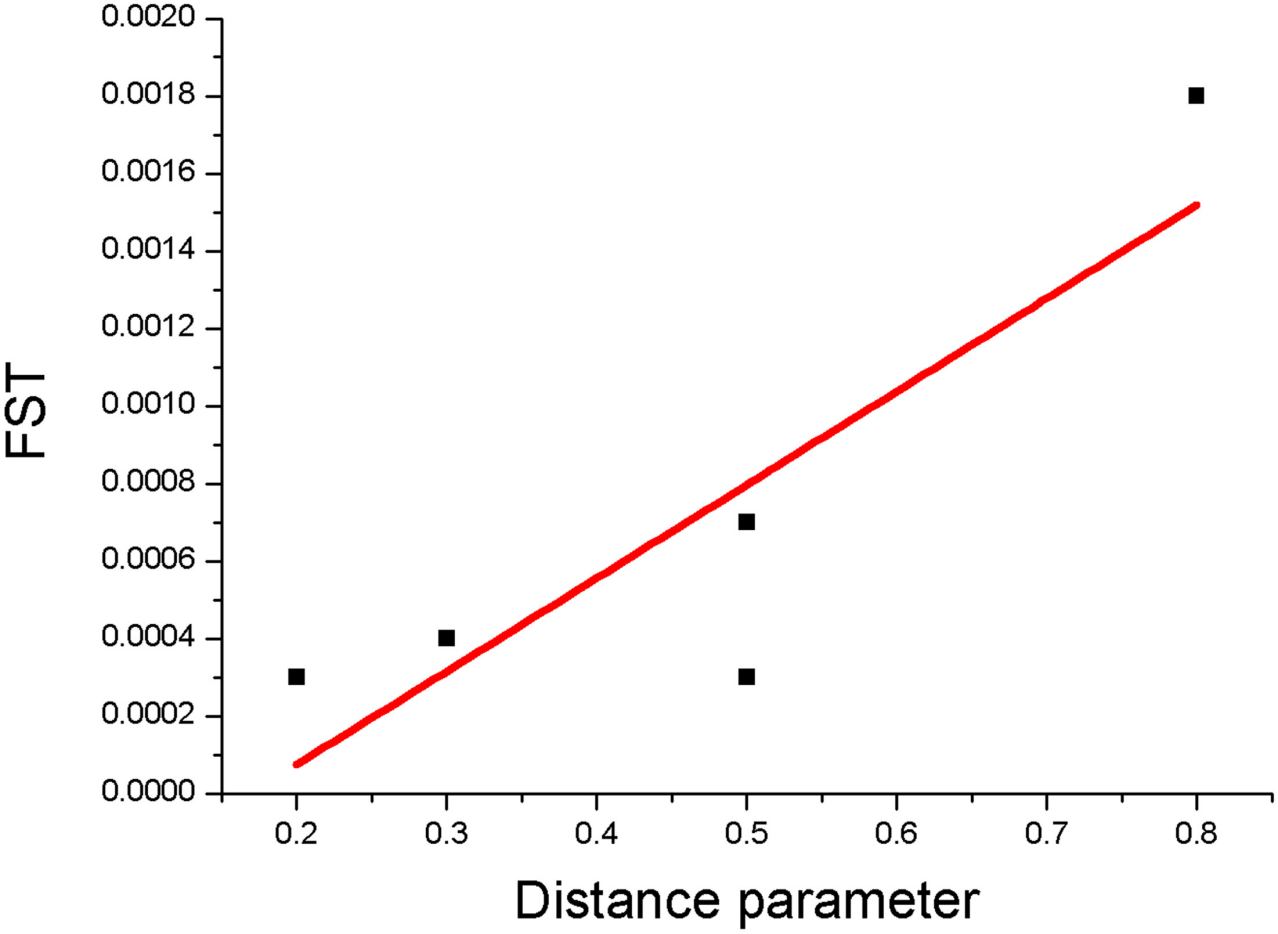
Comparison of the fixation index, F_ST_, deduced by Leslie et al. [7] in their genetic studies, with the distance parameter deduced from our population dynamics simulations. The Pearson correlation coefficient is 0.8703, indicating a strong positive correlation. The solid line is a spline fit to the data points and has a χ^2^ value of 0.7574.

In Fig 5 we compare the population density map of Great Britain (taken from Wikipedia) with genetic data [7] and our simulations. The population density map is well correlated with our map of land desirability, as would be expected. However, the comparison of genetic data with the simulation of the movement of people is more striking. While results of our simulations accurately reproduce the genetic groups, they also reveal an interesting pattern, that the people of North Central Britain travelled southward through a relatively inhospitable region while the population of southern Britain progressed through to the north far more consistently. This finding is in consonance with genetic data. In order to quantify the similarity that we qualitatively observe between genetic data and our results (Fig 5), we made use of the autosomal distance deduced by Leslie et al. [7]. In order to make appropriate correlations between the two data sets, we map our simulation results onto different geographical zones—North, Central North, East, and South West. We quantify the genetic distance between these regions on the basis of the number of steps that need to be computed in our simulations before populations from one region meet populations from another region. We denote this the distance parameter and assign a numerical value of 0 to the same population group (that is, interactions between populations within a single region); a numerical value of 1 implies maximum distance (interactions between populations that are geographically very widely separated). Our distance parameter is correlated (Fig 6) with the fixation index, F_ST_, used by Leslie et al. [7]. F_ST_ is a measure of population differentiation due to genetic structure and, at a given locus, is based on the variance of allele frequencies between populations.

Quantitative evaluation of correlations between two maps is accomplished by using Pearson’s correlation coefficient. The comparison we have made between the results from our population dynamics simulations and the genetic map [7] yields a Pearson correlation coefficient of 0.8703, indicating strong positive correlation. This comparison ignores the population in Cornwall which has very distinct genetic properties. The value of F_ST_ that includes the Cornish population yields a value [7] that is not inconsistent with the distance parameter deduced from our simulations.

This strong correlation has several important consequences. It shows that our relatively simple simulations, based on a particular combination of parameters, predict population dynamics which appear to be in good accord with a genetic map. Indeed, several detailed features agree very well, as enumerated in the following.

1. The current population map of the UK (Fig 4a) is in good agreement with the habitability of England, as calculated by our simulations (Fig 4c); population densities are well correlated to land habitability, confirming our basic assumption regarding the initial habitability of Britain.
2. The population of South Eastern England is the fastest moving of all populations; it rapidly comes in contact with all other groups. This is also consistent with the genetic data which suggest that a large fraction of genetic signal is of German or French origin; such people would, indeed, be expected to enter mainly through Southern England.
3. The Cornwall group remains confined to its narrow strait close to its point of origin even though it has a fairly large initial fraction of population (10^3^) compared to the initial South England population of 10^5^. The two groups merge close to the point where the geographical area widens to the East of Cornwall (Fig 3c). The genetic data for this region (Fig 4b—blue squares) also show the same results.
4. The Welsh population tends to migrate within present Welsh boundaries and does not move to Central England either in our simulations (Fig 3c) nor, indeed, in the genetic data (Fig 4b).
5. The population of Scotland remains bound to the highlands and the north English population also finds it hard to move northward (Fig 3b and 3c). The latter population makes much faster strides to the south, leaving the Scottish people isolated until much later. This is also reflected in the genetic data (see Fig 4c).
6. The population of North and South England tend to meet along the coast and slightly towards the central region. In this case, the simulation results differ slightly from the genetic data which tends to show the meeting of the two genetic groups further north.
7. The genetic data are similar to what our habitability data (Fig 4c) would suggest. We attribute this to a potential pinch effect. A small narrow path from North England towards South Central England—which was probably missed by the travellers but was exploited by the simulation—might have allowed faster movement of people.

It is pertinent to point out that the map that results from our computer simulations is also in consonance with the map of ancient habitation sites in Britain (see, for example, http://www.megalithic.co.uk/asb_mapsquare.php). It is noteworthy that appropriate choice of initial population also accurately reproduces the current population map.

To place our findings in perspective, we note that the idea of using diffusive modelling to understand movement of ideas and people in different populations has been used by various researchers [3, 4, 14, 15] to probe the relation of transport to population [3], to generalised stochastic aspects of migration [4], as well as diffusion between two population groups [16] and the migration of ideas across Europe [15]. Similar models specific to industrial growth have also been made to model industrialisation [17] which are of relevance from the perspective of economics [18]. The stability and efficiency of such simulations have also been analysed [18]. However, all such modelling is highly complex and incorporates far more parameters and complexity than the present problem requires.

Our simulation is designed to study the migratory pattern of *early* humans. We, therefore, do not assume their knowledge of farming nor of any other high-level skills. Such limitations actively discourage forming a large community. Consequently, the nature of our simulation actively discourages the formation of villages by not including accumulation of resources which might enhance habitability. Creation of large surplus values of resources is not permitted within our model. In order to include villages in our simulation we would need to model the resource generating capability of people [3], their inherent tendency to conglomerate into groups and we would have to account for the necessary increase in resources in a given area. All these parameters have hitherto been very poorly studied and any model requiring them would, of necessity, be highly subjective.

Our general purpose diffusive model of large scale human migration is sensitive to quantifiable and well understood parameters of water availability and flatness of land mass and altitude, as against more complex models of local area development [8] or the importance of waterways in Neolithic times [19].

The parameters used in our simulations are well constrained. We reiterate that the only parameters in the simulation are the distance from water source where people can survive, the population increase rate, and the initial populations. The simulation has a weak dependence on these parameters and, hence, the simulation is more or less deterministic, based on habitability (Fig 2). In particular, our simulation does not take into account soil fertility (except in the sense of water availability) and large scale temperature gradients.

In view of these considerations it not inappropriate for us to use a linear combination of parameters rather than a more complex (power law) dependence. While our results cannot define specific habitation centres—since we do not include the feature of human capacity for organisation—they do provide data on the general path travelled by the prehistoric populations. In turn, the simulation can yield fairly detailed information on where a small group might have traversed and then spread, giving rise to higher genetic uniformity. We, therefore, conclude that in spite of the nearly deterministic nature of the simulation it can be used to map prehistoric human migrations in some significant detail.

## Appendix

### Deducing parameters used in the simulation algorithm

We present here details of our simulation procedure and the choice of parameters we used.

**Fig 7:**
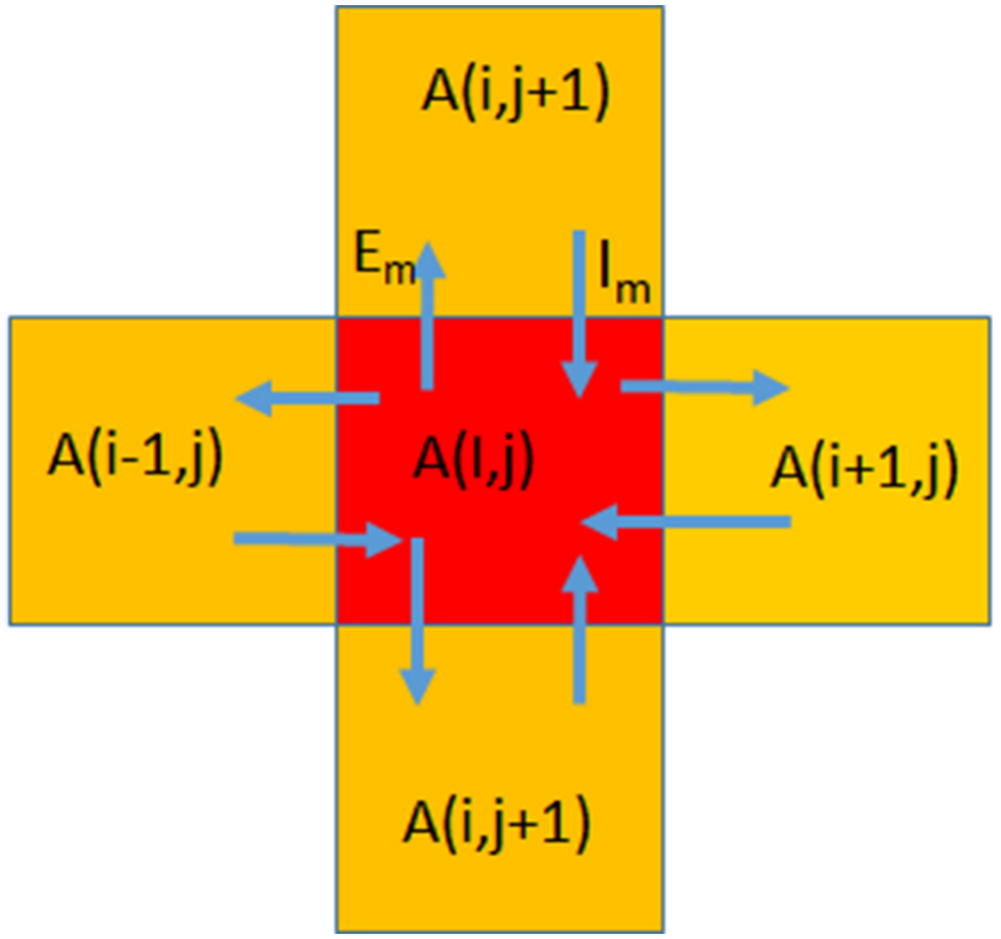
Basic units of computation. Each block was taken as 1 km by 1 km.

Our computations were carried out within boxes of dimension 1 km x 1 km. Within each box (Fig 7), we defined the following basic properties of a given box situated at a geographical location (i,j):

1. Altitude of the box A_ij_(i,j)
2. Altitude D_alt_(i,j)
3. Flatness D_surf_(i,j)
4. Proximity to water D_w_(i,j)
5. Population density

These five properties were used to deduce the derived property, the desirability R_in_(i,j) and, subsequently, the evaluated properties, relative desirability R(i,j).

The following parameters were then calculated:

1. Immigration rate I_m_(i,j)
2. Emigration rate E_m_(i,j)

From these, we evaluated the final value of the population, P(i,j).

### Description of basic parameters

#### Calculation of desirability as a function of altitude D_alt_(i,j)

Values of D_alt_(i,j) were evaluated according to the following:

$$D_{alt}(i,j)\;=\;\frac{1000-(7/3)\times A(i,j)}{1000}\;0<A(i,j)\leq 300$$

$$D_{alt}\left(i,j\right)\;=\;\left(\frac{\frac{A\left(i,j\right)-300}{9}+300}{1000}\right)\;3000<A\left(i,j\right)\leq 2100$$

$$D_{alt}(i,j)\;=\;\frac{\left(4100-A(i,j)\right)}{4\times 1000}\;2100<A(i,j)\leq 4100$$

$$D_{alt}(i,j)\;=\;\frac{1}{A(i,j)\times 1000}\;4100<A(i,j)$$

$$D_{alt}\left(i,j\right)=0\text{ A}\left(\text{i},\text{j}\right)<0$$

The constants and parameters were so adjusted to ensure that the local variations are highlighted.

#### Calculations of desirability as a function of flatness of land (D_surf_(I,j))

We defined the quality of land in terms of a parameter value, L_flat_. Land that was flatter than this value was considered excellent (while defining the rating of a location, see below) whereas land whose value of this parameter is 0.5 or lower was considered unliveable. We take A(i,j) as the altitude of the box at (i,j) as given by the original dataset. To estimate the flatness of a location, the mean altitude of all the 5 boxes is taken as A_mean_(I,j):

$$A_{mean}(i,j)\;=\;A(i-1,j)+A(i,j)+A(i+1,j)+A(i,j-1)+A(i,j+1)$$

We calculated an intermediate parameter, d_surf_(I,j), which was calculated using

$$d_{alt}(i,j)\;=\;\frac{abs(A_{mean}(i,j)-A(i,j))}{1000}\;A(i,j)\leq 300$$

$$d_{alt}(i,j)\;=\;\frac{abs(A_{mean}(i,j)-A(i,j))}{A(i,j)}300\;300<A(i,j)\leq 4100$$

$$d_{alt}(i,j)\;=\;0\;4100<A(i,j)$$

If d_surf_(i,j) was less than the flatness parameter (0.2), we defined

$$D_{surf}\left(i,j\right)\;=\;1-\left(\frac{1}{2\times L_{flat}}\right)\times d_{surf}\left(i,j\right)\;d_{surf}\left(i,j\right)\leq L_{flat}$$

$$D_{surf}(i,j)\;=\;\frac{L_{flat}}{2}\times d(i,j)\;L_{flat}<\;d_{surf}(i,j)\leq L_{tot}$$

$$D_{surf}(i,j)\;=\;0\;L_{tot}<\;d_{surf}$$

The logic behind this differentiation was to magnify local geographical variations while ensuring that the transition from sea level to high altitudes was smooth. If D_surf_(i,j) was less than L_flat_ (taken as 0.2) the land was assumed to be good from the viewpoint of habilitability. If it was between 0.2 and 0.5 it was assumed to be tolerable, and if it was greater than 0.5 the land was assumed to be too difficult to live on.

#### Calculation of desirability as a function of water availability (D_rive_)

We externally defined the radius (R) to which water from a water source would be easily potable. For each chosen site of an initial seed population this was an external parameter. For the present set of simulations, we used a uniform value of 10 pixels. We also defined R_m_ beyond which the water source was considered to be too far to be of practical utility. For the present simulation we took R_m_ = 50. For every water source we calculated the parameter S defined as

$$S\;=\;\sqrt{{\left(i-k\right)}^{2}\text{+}{\left(i-j\right)}^{2}}$$

We then calculated the parameter of water availability as:

$$D_{riv}(l,k)\;=\;1\;0<S\;\leq R$$

$$D_{riv}(l,k)\;=\;S^{-0.0435}\;R<S\;\leq R_{l}$$

$$D_{riv}(l,k)\;=\;0\;R_{l}<S$$

The final value of ${\text{D}}_{\text{riv }}\left(\text{i},\text{j}\right)=\overset{k\;=\;K}{\underset{k\;=\;1}{\sum }}\overset{l\;=\;L}{\underset{l\;=\;1}{\sum }}D_{riv}\left(i,j\right)$ where K and L are the total number of rows and columns in the simulation.

All these parameters were designed so that the parameter values varied between 0 and 1; the better the condition, the higher the value. Based on these values the absolute rating (R_in_(i,j)) of a location was established.

### Determination of absolute rating

If the land was located close to a water source, (D_riv_(i,j) > 0.5), and was flat (D_alt_(i,j) < L_flat_), then the rating was given by

$$R_{in}(i,j)\;=\;{D_{surf}(i,j)}^{2}\times D_{alt}(i,j)\times \;D_{riv}(i,j)$$

If the land was sloping within acceptable limits, (L_flat_ < D_alt_(i,j) < L_tot_) then the rating was calculated as

$$R_{in}(i,j)\;=\;{\left(D_{surf}(i,j)\times D_{alt}(i,j)\times \;D_{riv}(i,j)\right)}^{2}$$

For land on higher planes it was calculated as

$$R_{in}(i,j)\;=\;{\left(D_{alt}(i,j)\times D_{riv}(i,j)\right)}^{4}$$

The rating with poor water supply was calculated as

$$R_{in}(i,j)\;=\;{D_{alt}(i,j)}^{2}\times D_{surf}(i,j)$$

### Calculation of immigration and emigration rates

We defined P_max_ which is the maximum population possible anywhere. In the present simulation this was taken as 10^7^. We also defined a relative rating R(i,j) which took into account the independent rating R_in_(i,j) and the current population in the cell. This process is iterative. At any given time the immigration rate I_m_(I,j) and emigration rate E_m_(I,j) were determined by comparing with relative rating of the neighbouring cells, R(i-1,j), R(i+1,j), R(i,j-1) and R(i,j+1).

We defined the maximum local population P_o_(i,j) as

$$P_{o}(i,j)\;=\;P_{max}\times (0.5\;\times \;D_{surf}(i,j))+0.5\times D_{alt}(i,j)+\;D_{riv}(i,j))$$

Emigration rates (E_m_(i,j)) and Immigration rates (I_m_(i,j)) were based on the difference in rating and P_max_. For each box, the R_in_ is a comparison of rating of a cell (i,j) with its neighbouring cell (l,m) where l varies from i-1 to i+1 and m from j-1 to j+1.

We calculated the emigration and immigration along the two dimensions separately. Consider a cell with population P_0_(i,j) which has a relative rating R(i,j), with the population-independent rating being R_in_(i,j).

The population that wants to migrate is as indicated below. In all the equations given below, in order to avoid making the equations long, we use the symbol ± to indicate two terms, one for x+y and the other for x-y and does not include the term (x,y).

$$P(i,j)\;=\;P_{0}(i,j)\times (1-R(i\pm 1,j)\times R(i\pm 1,j\pm 1)\times R(i,j))$$

This sum has 4 terms in all.

We defined β as the fraction of people who want to migrate in the horizontal direction.

$$\beta (i)\;=\;\frac{P(i-1,\;j)+P(i+1,j)}{R(i-1,j)+r(i+1,j)}$$

$$\beta (j)\;=\;\frac{P(i,j-1)+P(i,j+1)}{R(i,j-1)+R(i,j+1)}$$

Given a direction in cell i, f(i) is the fraction of people wanting to emigrate through boundary i. So the fraction of population that tries to emigrate through boundary i is

P(i,j) = (P(i-1,j)+P(i+1,j))×β(i)+(P(i,j-1)+P(i,j+1))×β(j)

We then determined the flux F_in_ as the fraction of P that successfully migrates across the boundary. F_out_ is the fraction of P(I,j) that immigrates

$$\frac{F_{in}}{F_{out}}\;=\;\frac{R(i+1,j)}{R(i,j)}$$

We normalized F_in_ + F_out_ = 1

Therefore,

$$I_{m}(i,j)\;=\;F_{in}\times P(i,j)\;=\;F_{in}\times P\times \beta (i)\times \beta (j)$$

and,

$$E_{m}(i)\;=\;F_{out}(P(i\pm 1,j)\times \beta (i\pm 1)+P(i,j\pm 1)\times \beta (i,j\pm 1))$$

For each box E_m_ and I_m_ from the four neighbouring boxes were added to determine the net migration into a region. This was then compared with the maximum population a location can handle. As the population increases, two quantities were calculated as follows:

$$S_{1}(i,j)\;=\;(\sqrt{R_{in}(i,j)}{-R}_{in}(i,j))/P_{b}$$

$$S_{2}(i,j)\;=\;\sqrt{R_{in}(i,j)/P_{b}}$$

If the population in a region was less than the maximum population, the rating was changed as:

$$R_{i}(i,j)\;=\;R_{in}(i,j)+S_{1}\times P(i,j)$$

Where P(i,j) is the current population in the cell (i,j)

If the population exceeded, P_max_, but was less than twice the maximum, we calculated

$$R_{j}(i,j)\;=\;P(i,j)-2\;\times P_{b}(i,j)\times S_{2}(i,j)$$

In case the population exceeded twice the maximum population, the emigration rate was increased to bring the population down by setting the rating to 0.

This was then fed back into the calculation to calculate the total population in each cell and the relatice rating. This permited a certain amount of local spike in population to allow for stabilization. It also discouraged city formation by transferring larger populations away from cells that were already highly populated.
